# Vaccination status, incidence of adverse events, and awareness of COVID-19 vaccine among outpatients undergoing chemotherapy

**DOI:** 10.1186/s40780-024-00338-w

**Published:** 2024-03-04

**Authors:** Satoshi Iwakawa, Takuya Azechi, Orie Saigo, Ryunosuke Imai, Ayumi Nakai, Shoji Koshiba, Uki Saito, Kota Asakura, Kuniyoshi Sato, Toshimi Kimura

**Affiliations:** Department of Pharmacy, Juntendo University Hospital, 3-1-3 Hongo, Bunkyo-ku, Tokyo, Japan

**Keywords:** COVID-19 vaccination, Adverse events, Chemotherapy, Consultation channels, Information sources

## Abstract

**Background:**

Cancer has been identified as a risk factor for severe illness and mortality in coronavirus disease (COVID-19), underscoring the importance of recommending COVID-19 vaccinations to patients with cancer. However, few reports have focused on the vaccination status and the incidence of adverse events among patients with cancer. In this study, we aimed to evaluate the vaccination status, incidence of adverse events, concerns, and anxiety related to COVID-19 vaccination among patients with cancer. In addition, we explored the utilization of information sources by these patients and the ease of use.

**Methods:**

A survey was conducted among outpatients undergoing chemotherapy who received medication counseling from a pharmacist at Juntendo University Hospital. Responses were gathered from 60 out of the 143 participants. Of the respondents, 96.7% had received two doses of the COVID-19 vaccine.

**Results:**

Common adverse events included pain at the injection site, fever, and fatigue, which were experienced by nearly half of the respondents. Approximately 80% expressed some concern regarding vaccination, with predominant concerns about timing in the context of ongoing cancer treatment and surgery. Among the respondents, 41.7% consulted primary care physicians regarding the vaccine, with only one mentioning consultation with hospital pharmacists. Notably, primary care physicians were considered the most approachable and useful healthcare professionals.

**Conclusions:**

These results suggest that patients with cancer can safely receive the vaccine, comparable to patients without cancer. However, they still harbor concerns, even when seeking advice from primary care physicians. Few patients consulted pharmacists about vaccination, highlighting an opportunity for pharmacist intervention. Pharmacists fostering trust with patients with cancer is imperative to explore pharmacist intervention methods to promote the continued administration of COVID-19 vaccines and enhance the quality of life for them.

**Supplementary Information:**

The online version contains supplementary material available at 10.1186/s40780-024-00338-w.

## Background

Vaccination contributes to reducing the mortality and incidence rates of various infectious diseases, such as eradicating smallpox, representing one of the greatest achievements in public health [1]. In the context of coronavirus disease (COVID-19), in addition to preventive measures centered on standard precautions, the development and widespread administration of vaccines are essential to prevent the spread of the disease [2]. Since the approval of COVID-19 vaccines in February 2021, Japan has employed several of these vaccines [3–5]. Although vaccination has decreased the risk of infection and severe illness, the emergence of mutant viruses has fueled the persistence of COVID-19. In May 2022, a fourth dose of the vaccine was administered to older people and individuals with underlying health conditions. The fifth dose of the vaccine was initiated in September 2023, indicating the need for continuous COVID-19 vaccine administration [6].

Because cancer is cited as a risk factor contributing to the mortality and severity of COVID-19, vaccination is recommended for patients with cancer [7, 8]. However, few reports have focused on the vaccination status and the incidence of adverse events among patients with cancer. Concerns about vaccine-related adverse events, uncertainties regarding the efficacy and safety of vaccines, and skepticism about the rapid development of vaccines constitute major reasons for hesitancy toward COVID-19 vaccination [9]. When patients with cancer experience anxiety or concerns about vaccination, they access various sources of information to address their concerns. Because incorrect information may be obtained depending on the information source, the involvement of healthcare professionals is important to alleviate these concerns. However, there have been insufficient reports on anxiety and concerns in patients with cancer, the information sources they access, and their interactions with healthcare professionals.

In this study, we aimed to clarify the vaccination status and the incidence of adverse events related to COVID-19 vaccination among patients with cancer. In addition, we elucidated the anxiety and concerns of patients with cancer regarding COVID-19 vaccination, consultation channels, information sources before vaccination, and their ease of utilization among patients with cancer.

## Materials and methods

### Study design and participants

This study was a cross-sectional, web-based, anonymous questionnaire survey designed to clarify the COVID-19 vaccination rate, adverse event occurrences, anxiety and concerns about COVID-19 vaccination, usage and ease of consultation channels, and information sources among patients with cancer. This survey was conducted among outpatients undergoing chemotherapy who received medication counseling from the pharmacist at Juntendo University Hospital from October 1, 2022, to January 31, 2023. In this hospital, five pharmacists with specialized or certified qualifications related to cancer treatment are employed at a time. Among them, one pharmacist is dedicated daily to providing medication counseling services to outpatients with cancer. After the pharmacist provided information regarding the purpose of the survey and guidance on the online questionnaire was given, participants accessed the survey. The details of the survey items are shown in Additional file 1. Each survey item was collectively devised based on multiple information sources and reports concerning adverse events [4, 5, 10, 11] by the co-authors who are experienced and certified pharmacists specializing in cancer chemotherapy. Thereon, the questionnaire was finalized after consultation with pharmacists specialized in infection control.

### Data analysis

Data were collected using Questant software, an online questionnaire program (Macromill Inc., Tokyo, Japan). Simple tabulation and statistical analysis were performed using the JMP pro 17 (SAS Institute, Cary, NC, USA). A scoring system was implemented to compare the ease of utilizing consultation channels and information sources as a reference for COVID-19 vaccination. Each answer was given a specific score; “Very easy to consult/utilize” = 5, “Easy to consult/utilize” = 4, “Neutral” = 3, “Difficult to consult/utilize” = 2, “Very difficult to consult/utilize” = 1, and " Not involved” = 0. The averages and standard deviations for each item were calculated. To compare the average value of each item with “hospital pharmacist” as the control, a Dunnett’s test was performed. Statistical significance was set at a *P* level of < 0.05.

## Results

### Respondent background and the reasons for non-vaccination

The response rate to the questionnaire was 42.0% (60/143 patients undergoing chemotherapy). The demographic and clinical characteristics of the participants are summarized in Table 1 and Additional file 2. Notably, 97% of the respondents received two doses of the COVID-19 vaccine. Moreover, 21.7% of the respondents contracted COVID-19 after receiving the vaccine. Table 2 lists the reasons for non-vaccination. A common response for missing the third and fourth vaccine doses was “Schedule did not match.” Additionally, for missing the fourth dose, the most frequently received response pertained to “Interaction with current medications.” In addition, the “Others” category included responses indicative of plans for future COVID-19 vaccination.

**Table 1:** Demographic and clinical characteristics of the participants

		Number	%
Sex	Male	16	26.7
	Female	44	73.3
Age (years)	< 40	3	5.0
	40–49	15	25.0
	50–59	21	35.0
	60–69	13	21.7
	70–79	8	13.3
	≥ 80	0	0.0
History	Allergy	9	15.0
	Experience of feeling unwell after receiving some vaccine	5	8.3
Vaccination status	Never	2	3.3
	1st dose	58	96.7
	2nd dose	58	96.7
	3rd dose	47	78.3
	4th dose	21	35.0
Cancer stage	Stage 0	0	0.0
	Stage I	9	15.0
	Stage II	13	21.7
	Stage III	13	21.7
	Stage IV	13	21.7
	Unknown	12	20.0
Duration of cancer treatment	< 1 years	44	73.3
	1–5 years	9	15.0
	6–10 years	5	8.3
	≥ 11 years	2	3.3
History of COVID-19	Have a history of COVID-19 without the vaccination	0	0.0
	Have a history of COVID-19 before vaccination	2	3.3
	Have a history of COVID-19 after vaccination	12	20.0
	Have a history of COVID-19 both before and after vaccination	1	1.7
	No history of COVID-19	45	75.0

COVID-19, coronavirus disease

**Table 2:** Reasons for non-COVID-19 vaccination and interruption of COVID-19 vaccination

	1st dose		
Concerns about safety, such as severe adverse events	1		
Unknown evidence about its effectiveness	0		
Unknown relief services for adverse health effects	0		
Unknown medical procedure for a severe allergic reaction	0		
No habit of vaccinations such as flu	0		
Not recommended by my friend	0		
Unable to receive vaccination due to allergy status	0		
Chronic disease	0		
Interaction with current medications	0		
Schedule did not match	1		
Others	0		
	2nd does	3rd dose	4th dose
The adverse events were intolerable	0	2	1
I did not feel the effectiveness of the vaccination	0	0	0
I had allergic symptoms	0	0	0
Vaccination impaired physical and mental health	0	1	1
Not recommended by my friend	0	1	0
Chronic disease	0	1	0
Interaction with current medications	0	1	7
Schedule did not match	0	4	4
Others	0	4	13

Numbers in the table indicate the number of respondents

COVID-19, coronavirus disease

### Basic knowledge and awareness of COVID-19 vaccination

Table 3 shows the results of the responses regarding basic knowledge and awareness of COVID-19 vaccination. A significant 72% of the respondents believed that COVID-19 vaccines alleviate symptoms of COVID-19, and no respondents answered: “COVID-19 vaccines completely prevent COVID-19.” Nearly half of the respondents expressed concerns about ongoing cancer treatments or surgeries and questioned the appropriate timing for vaccination. Furthermore, 20% of the respondents answered “no specific concerns.”

**Table 3:** ReasonsBasic knowledge and awareness of COVID-19 vaccination in cancer patients

		Number	%
COVID-19 vaccine’s efficacy	COVID-19 vaccines completely prevent COVID-19	0	0.0
	COVID-19 vaccines are highly effective in preventing COVID-19	28	46.7
	COVID-19 vaccines tend to alleviate the symptoms of COVID-19	43	71.7
	Vaccination provides sustained protection against COVID-19–associated serious complications	24	40.0
	Mass infections (cluster infections) can be prevented by the vaccination	9	15.0
	Nothing applies	4	6.7
Concerns regarding COVID-19 vaccine	May be ineffective or poor response because of cancer	1	1.7
	Cancer may get worse	11	18.3
	COVID-19 vaccines may cause COVID-19 in patients with weekend immune system	15	25.0
	When should I get vaccinated if I am scheduled for cancer treatment or surgery?	29	48.3
	Probability of cancer-specific adverse events	24	40.0
	Increasing adverse events than the other people such as pain at the vaccination site	11	18.3
	Possibility of vaccination for the people in the developed an allergy to chemotherapy	0	0.0
	Possibility of reducing the effectiveness of cancer treatment by vaccination	7	11.7
	Possibility of interruption of cancer chemotherapy	13	21.7
	Which vaccine should I choose?	4	6.7
	Are the vaccines safe?	14	23.3
	No specific concerns	12	20.0

COVID-19, coronavirus disease

### Adverse events of COVID-19 vaccination

Table 4 shows the profiles of adverse events of COVID-19 at each dose. Irrespective of the dose, the most prevalent adverse event was localized pain at the injection site. Regarding systemic symptoms, fever, and fatigue rates were higher than other symptoms at each dose. Most respondents reported that their schedules for cancer treatment were unaffected.

**Table 4:** COVID-19 vaccination profile and adverse events in cancer patients

		1st dose			2nd dose			3rd dose			4th dose	
		Number	%		Number	%		Number	%		Number	%
Type of vaccinations	Pfizer	43	74.1		46	79.3		28	59.6		15	71.4
	Moderna	14	24.1		11	19.0		19	40.4		6	28.6
	AstraZeneca	0	0.0		0	0.0		0	0.0		0	0.0
	Others	0	0.0		0	0.0		0	0.0		0	0.0
	I do not know /I do not remember	1	1.7		1	1.7		0	0.0		0	0.0
	Not answer	0	0.0		0	0.0		0	0.0		0	0.0
Impact on cancer treatment schedule	Yes	3	5.2		0	0.0		1	2.1		1	4.8
	No	54	93.1		56	96.6		44	93.6		19	90.5
	Not answer	1	1.7		2	3.4		2	4.3		1	4.8
Presence of adverse events	Shock/Anaphylaxis	0	0.0		0	0.0		0	0.0		0	0.0
	Pain at the injection site	23	39.7		21	36.2		18	38.3		6	28.6
	Swelling at the infection site	10	17.2		10	17.2		9	19.1		4	19.0
	Redness/erythema at the injection site	1	1.7		2	3.4		1	2.1		1	4.8
	Headache	5	8.6		5	8.6		2	4.3		1	4.8
	Diarrhea	0	0.0		0	0.0		0	0.0		0	0.0
	Muscle pain	10	17.2		10	17.2		6	12.8		1	4.8
	Joint pain	6	10.3		6	10.3		6	12.8		1	4.8
	Fatigue	13	22.4		13	22.4		10	21.3		3	14.3
	Cold	5	8.6		5	8.6		6	12.8		0	0.0
	Fever (over 37.5ºC)	21	36.2		17	29.3		13	27.7		3	14.3
	None	27	46.6		28	48.3		24	51.1		14	66.7

COVID-19, coronavirus disease

### Consultation channels and information sources used for COVID-19 vaccination

**Fig. 1:**
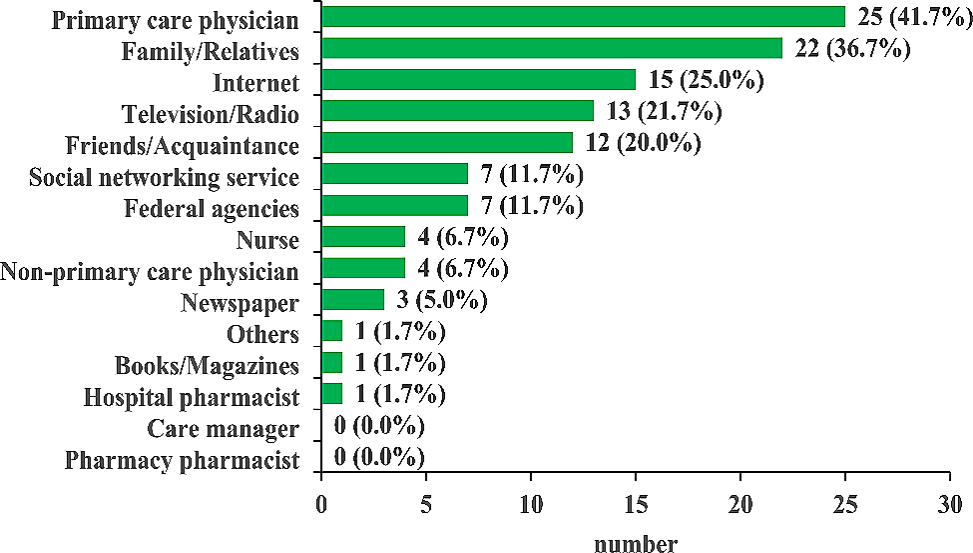
Utilization patterns of consultation channels and information sources

Figure 1 shows the utilization pattern of consultation channels and information sources related to COVID-19 vaccination. The top three channels/sources with notably high response rates as consulted references were “primary care physician,” “family/relatives,” and “Internet.” No responses indicated consultation with a “pharmacy pharmacist,” and only one respondent consulted a “hospital pharmacist.” Table 5 shows the ease of utilizing consultation channels and information sources related to COVID-19 vaccination. When ranking the ease of utilization for each consultation channel or information tool, “family members/relatives” were most accessible, which was statistically higher than “hospital pharmacists.” Furthermore, among healthcare professionals, “primary care physicians” were the most accessible; however, no statistically significant difference was observed in the average compared to “hospital pharmacists.” On the other hand, “pharmacy pharmacists” displayed a tendency towards lower average value compared to “hospital pharmacists,” although no statistically significant difference was noted.

**Table 5:** Ease of utilizing consultation channels and information sources

Consultation channels and information sources	Ease of utilizing consultation channels and information sources							Average score	Standard deviation	p-value*
	Very easy	Easy	Neutral	Difficult	Very difficult	Not involved				
Hospital pharmacist	16	12	14	0	1	17		2.85	1.98	-
Pharmacy pharmacist	9	7	20	1	1	22		2.27	1.90	0.381
Primary care physician	25	14	10	1	0	10		3.55	1.78	0.184
Non-primary care physician	8	10	20	4	2	16		2.50	1.76	0.911
Nurse	21	11	13	2	0	13		3.20	1.89	0.911
Care manager	5	2	5	0	0	48		0.80	1.67	< 0.001
Family/Relatives	31	17	11	1	0	0		4.30	0.83	< 0.001
Friends/Acquaintance	14	20	15	1	2	8		3.32	1.60	0.660
Federal agencies	3	4	23	2	0	28		1.73	1.72	0.004
Television/Radio	2	4	29	1	1	23		1.93	1.62	0.031
Newspaper	1	4	22	2	1	30		1.53	1.62	0.000
Books/Magazines	1	5	24	0	1	29		1.63	1.66	0.001
Internet	6	13	24	1	1	15		2.62	1.69	0.996
Social networking service	4	7	23	3	2	21		2.08	1.71	0.113

* Statistical differences compared to Hospital pharmacist as a control were determined by Dunnett’s test

## Discussion

Several surveys in the general population have shown that the frequency of adverse events of COVID-19 vaccine, such as fever and fatigue, increased after the second dose compared with the first dose [4, 12]. However, we found that the incidence of fever after the first dose in patients with cancer was higher than that in a previous report and was almost the same as that of the second vaccination. Although the individual clinical course of the respondents is unknown because of the anonymous questionnaire used in this study, factors other than vaccine-related adverse events, such as fever with febrile neutropenia after chemotherapy, might be involved. It is difficult to distinguish between these factors that lead to fever after vaccination; since febrile neutropenia necessitates urgent intervention, patients with cancer need to know what to do if they have a fever after receiving the COVID-19 vaccination.

COVID-19 vaccination is recommended for patients with cancer because of the high risk of mortality and the severity associated with a compromised immune response during chemotherapy [7, 8]. A systematic review and meta-analysis reported that female sex and chemotherapy were significant factors associated with COVID-19 vaccination hesitancy in patients with cancer [9]. However, most of the respondents in this study had received two or more doses of the COVID-19 vaccine at the time of their responses, demonstrating a vaccination rate higher than the overall vaccination rate in Japan [13]. Over half of the respondents acknowledged the infection prevention and severity mitigation effects of COVID-19 vaccination, leading them to believe in the vaccine’s efficacy and receive it. Additionally, 41.7% of the respondents cited their “primary care physician” as the consultation source, suggesting that communication with physicians might have influenced their decision to be vaccinated.

Regarding the effectiveness of the COVID-19 vaccination, some respondents indicated a lack of correct understanding of the low response rates for herd immunity (15%) and a misunderstanding of the potential cause of COVID-19 post-vaccination (25%). These findings suggest that the respondents did not understand the efficacy of COVID-19 vaccination in patients with cancer. Continuous dissemination of information regarding the utility of COVID-19 vaccination in patients with cancer is crucial.

Even after vaccination, it is well known that people continue to have some anxiety and concern about the vaccine [14]. This survey also confirmed that 80% of the respondents had anxiety or concerns about COVID-19 vaccination. Regarding the timing of COVID-19 vaccination that showed the highest response as a concern in this study and other reports [15], the Japanese Society of Clinical Oncology recommends the time period when it is better to avoid COVID-19 vaccination [8]. Considering the latest domestic and international evidence, information regarding vaccination timing is deemed beneficial for endorsing the COVID-19 vaccination. Furthermore, approximately 20–40% of the respondents in this study expressed concerns about the incidence of adverse events, unique adverse events, and the safety of the COVID-19 vaccine. Given that concerns about vaccine-related adverse events can be a deterrent to vaccination [16], providing healthcare professionals with information on adverse events is crucial. Compared with other survey reports [4, 12, 17], this survey found no higher incidence of adverse events or unique adverse events regardless of the number of COVID-19 vaccine doses administered, except for a tendency toward more frequent fevers after the first dose. Additionally, this study has identified concerns of patients with cancer regarding COVID-19 vaccine interactions with current medications, and post-vaccination fever in relation to COVID-19 vaccination. Generally, while antipyretics are the first choice for fever management after vaccination, fever following chemotherapy may require appropriate intervention, and the approach may vary. However, our institution lacks guidelines pertaining to fever management. Additionally, although there are risks such as bleeding at the administration site due to antithrombotic agents, there are no explicit recommendations regarding interactions with concomitant medications. Patients undergo vaccination based on the discretion of their attending physicians. To potentially enhance vaccination rates, it may be necessary to establish institutional regulations concerning fever management and interactions between the vaccine and current medicines.

Additionally, there was no difference in the occurrence of adverse events related to COVID-19 vaccination based on the presence or absence of chemotherapy [18]. While many uncertainties remain regarding the long-term safety of COVID-19 vaccination, these findings suggest that patients with cancer can safely receive the vaccine, similar to healthy individuals. Communicating this lack of significant differences in adverse reactions between patients with cancer and the general population can be valuable in allaying concerns. Furthermore, this survey obtained responses citing the severity of adverse events and health damage as reasons for interrupting COVID-19 vaccination. For patients with cancer, it is necessary to assess the feasibility of continuing COVID-19 vaccination from a medical and pharmaceutical perspective, along with implementing interventions, such as proposing medications for symptom relief, to enhance vaccination rates.

Prospective vaccine recipients gather and evaluate information from various sources to address their anxieties and concerns. Telephone interview surveys showed that physicians (77.6%) and federal agencies (50.5%) were highly trusted sources of information on the COVID-19 vaccine, whereas family members (25.9%) and social media (3.8%) were deemed less reliable [19]. People relying on family/friends, faith-based organizations, or social media for healthcare information tend to be hesitant about getting vaccinated [20]. However, even with these information sources considered to be unreliable, our study indicated a high percentage of respondents who utilize “family/acquaintance” and “social networking service” as the source of consultation and information. Conversely, workplaces/schools, LINE (a communication tool like WhatsApp and iMessage highly popular in Japan), and social media sources have encouraged citizens to receive the COVID-19 vaccine [21]. Although the impact of information sources on vaccination intention was not thoroughly examined in this study, it is plausible that consultations with family/friends and the use of social media could have contributed to the high COVID-19 vaccination rates. Continuous research is essential to understand the influence of various information sources on patients with cancer who intend to receive the COVID-19 vaccine.

Consultation with “primary care physicians” was the most common response in this study. This underscores the significance of primary care physicians for patients with cancer in making decisions regarding vaccination feasibility, considering the impact on cancer treatment, vaccination schedules, and existing health conditions. Notably, only one respondent mentioned pharmacists in the survey, indicating a perception of no involvement of pharmacy/hospital pharmacists compared to other healthcare professionals, making them less utilized as consultation channels, possibly because of limited communication opportunities compared to other professions. Particularly in our institution, pharmacist’s interventions for the patients with cancer are primarily focused on the initial and second treatments, with limited opportunities for sustained interventions. Consequently, there are few occasions for pharmacists to engage in communication with the patients with cancer, potentially resulting in lower contact with pharmacists compared to other healthcare professionals. In addition, the low response rate of 42.0% in this study can be attributed to the scarcity of communication opportunities with pharmacists, along with the possibility that some elderly individuals are less proficient in operating tablet devices such as smartphones. Consultations with primary care physicians may have resolved patient’s concerns about COVID-19 vaccination, making consultations with other healthcare professionals unnecessary. However, some respondents in this study still harbored concerns about the COVID-19 vaccination, indicating that unresolved anxieties and worries might persist despite consulting primary care physicians.

In this survey, “hospital pharmacist” was among the easiest to approach, following primary care physicians and nurses. This could be attributed to the trust established through medication counseling and adverse event monitoring during outpatient chemotherapy. With the promotion of designated pharmacies and advanced pharmaceutical management functions, pharmacists are expected to build better relationships with patients with cancer, enhancing the ease of consultation. Pharmacists with an established trust could potentially address the unresolved concerns of patients with cancer, proving invaluable for the continued promotion of COVID-19 vaccination. Since utilization of our hospital as a consultation point is limited, we believe that fostering a trustful relationship with patients with cancer is crucial. This can be achieved by pharmacists conducting sustained interventions and increasing opportunities for communication with cancer patients. A detailed exploration of patient anxieties regarding the COVID-19 vaccine and assessment of pharmacist interventions is crucial for future research.

The limitation of this study may impose difficulty in generalizing our findings and conclusions. The survey was conducted within a single hospital, and the sample size was small. Additionally, there is a significant bias towards cancer types (shown in an Additional file 2), and the participants in this survey may not fully represent the entirety of patients with cancer. Moreover, we did not confirm the treatment regimens and the timing of chemotherapy of patients with cancer. Therefore, the effects of these factors on the purpose of this study could not be evaluated. Although definitive conclusions are challenging, valuable insights were gained to consider the necessary interventions to foster the ongoing acceptance of COVID-19 vaccine administration among patients with cancer.

## Conclusions

The high vaccination rate among patients with cancer indicates that they can receive the COVID-19 vaccine with the same level of safety as patients without cancer. Furthermore, while patients with cancer engage in COVID-19 vaccination consultations primarily with their primary care physicians, they may harbor some form of apprehension. However, access to pharmacists for administering the COVID-19 vaccine to patients with cancer is limited, highlighting the potential for pharmacist intervention. It is essential to explore pharmacist intervention methods to promote continuous vaccination against COVID-19, thereby maintaining and enhancing the quality of life of patients with cancer.

### Electronic supplementary material

Below is the link to the electronic supplementary material.

Additional file 1: Survey items

Additional file 2: Cancer type and use of immune checkpoint inhibitor of the respondents

## Data Availability

All data generated or analyzed during this study are included in this published article.

## Abbreviations

(COVID-19): coronavirus disease
